# Commonalities and differences in healthcare workers’ perceptions of mental burden in Brazil, Colombia, and Germany during the COVID-19 pandemic: a qualitative cross-country study

**DOI:** 10.3389/fpubh.2025.1542494

**Published:** 2025-07-15

**Authors:** Andrea Tenorio Correia da Silva, Felix Sisenop, Alexandra Restrepo Henao, Miriam Regina Souza, Beatriz Atti, Maria Paula Ruiz, Evaldo Marchi, Jutta Lindert

**Affiliations:** ^1^Department of Primary Care, Santa Casa de São Paulo School of Medical Sciences, São Paulo, Brazil; ^2^Department of Social Work and Health, University of Applied Sciences Emden/Leer, Emden, Germany; ^3^Epidemiology Research Group, University of Antioquia, Colombia, and Postdoctoral Researcher, The City University of New York, New York, NY, United States; ^4^Faculty of Medicine of Jundiaí, Jundiaí, Brazil

**Keywords:** cross-country study, mental health, psychological distress, healthcare workers, community health workers, COVID-19, misinformation, workplace violence

## Abstract

**Objective:**

To assess healthcare workers’ (HCWs) perceptions of commonalities and differences in stressors affecting their mental health during the COVID-19 pandemic in Brazil, Colombia, and Germany.

**Method:**

We conducted a qualitative cross-country study between April and September 2022. HCWs from primary and secondary care centers and hospitals participated. The focus groups were recorded, transcribed verbatim, and analyzed using content analysis.

**Results:**

63 HCWs participated in the study. Factors affecting HCWs’ mental health were categorized into three levels: healthcare system, facility, and individual. Common stressors across all three countries included: a rapid increase in patients and disease severity at the healthcare system level; frequent updates to COVID-19 protocols, staff shortages, and service unpreparedness at the facility-level; and dealing with a new virus and workplace violence at the individual-level. In Brazil and Colombia, constraints in the healthcare system and insufficient personal protective equipment were reported. Additionally, in Brazil, financial strain and working in deprived areas also impacted HCWs’ mental health.

**Conclusion:**

Improving healthcare system preparedness, reducing inequities across facilities, and combating misinformation and violence are crucial for alleviating the mental burden on HCWs in future global crises.

## Introduction

The COVID-19 pandemic introduced numerous stressors that significantly impacted the physical and mental health of healthcare workers (HCWs) worldwide (1, 2). However, these stressors were not experienced uniformly across countries. They were shaped by contextual factors such as healthcare systems structures, resources availability, and governmental responses to the crisis (3–5). Understanding these variations is critical for developing support strategies that are both globally informed and locally responsive. Cross-country comparisons are therefore critical to identifying both shared and context-specific mental health challenges faced by HCWs.

A recent meta-analysis indicates high prevalence rates of anxiety (37%), depression (36%), and insomnia (32%) among HCWs during the pandemic (6). While most studies have focused on hospital-based staff, particularly those in emergency departments and intensive care units, primary care workers have received considerably less attention (2). This represents a significant gap, given their central role in pandemic response, serving as the first point of contact and ensuring continuity of care (7) for most patients during the pandemic. They were responsible for early screening, public health education, monitoring of vulnerable populations, and continuity of care—all under challenging conditions. As such, primary care workers likely faced unique stressors, including increased community exposure, limited resources, and evolving responsibilities, which may differ substantially from those encountered in secondary or tertiary care settings.

Previous multi-country qualitative studies have explored changes in primary care during the pandemic across in various regions such as Africa, Asia, Europe and the Americas (8–10). However, many of these studies focused on a single geographic region, lacked systematic cross-country analysis, or relied on brief open-ended survey responses rather than in-depth interviews. Moreover, a recent scoping review highlighted that most qualitative research on HCWs’ mental health was conducted in high-income countries. These studies revealed stressors at multiple levels-individual (e.g., emotional exhaustion, disrupted professional identity), interpersonal (e.g., strained family and colleague relationships), and institutional levels (e.g., lack of organizational support, policy uncertainties) (11).

This study focuses on Brazil, Colombia, and Germany—three countries with distinct healthcare systems and contrasting governmental responses to the COVID-19 pandemic. Brazil has a universal, publicly funded system (12); Colombia operates a mixed model based on private insurance and public subsidies; and Germany’s system is financed through employer and employee contributions (13). As of July 2024, Brazil had reported 37.5 million confirmed cases, Colombia 6.4 million, and Germany 38.4 million (14). These variations reflect differences in health infrastructure as well as divergent political and public health strategies—ranging from denial and misinformation in Brazil to evidence-based policies in Colombia and Germany. These contextual contrasts present a unique opportunity to explore how different national approaches influenced HCWs’ mental health.

This study examines HCWs’ perceptions of mental health burden in Brazil, Colombia, and Germany. We focused specifically on primary care workers, given their strategic role in pandemic response and their relative underrepresentation in existing research. By conducting qualitative interviews using a common methodology across the three countries, we aim to identify both shared and context-specific stressors that shaped HCWs’ mental health experiences. These findings also inform hypotheses for a subsequent quantitative study. To our knowledge, this is the first qualitative investigation to explore HCWs’ perceptions of mental health of across Latin America and Europe using a cross-country comparative design. By interviewing primary care workers in Brazil, Colombia, and Germany - countries with markedly different healthcare infrastructures, pandemic trajectories and burden of disease and mortality - our study fills this critical gap by offering insights into HCWs’ mental health. Given the strategic role of primary care in managing the pandemic, a focused exploration of primary care workers’ mental health experiences is essential for strengthening healthcare system resilience in future crises.

## Method

### Study design and participants

We conducted a cross-country qualitative study with purposively selected participants from primary care centers in Brazil, primary, secondary, and tertiary care facilities in Colombia, and hospitals in Germany. In Brazil, we included 45 physicians, nurses, nursing assistants, administrative staff, community healthcare workers (CHWs), and other clinical staff working in primary care during the pandemic in São Paulo. In Colombia (*n* = 9) and Germany (*n* = 9), we included doctors, nurses, and auxiliary nurses. Exclusion criteria included not having worked during the pandemic or being on sick leave at the time of the interview.

### Procedures

We conducted focus groups—four in Brazil, three in Colombia, and three in Germany—and performed a content check. Participants were selected to represent a range of roles and settings, contacted via facility leaders, to ensure diverse perspectives.

A interview guide was originally developed in English and then translated into Portuguese, Spanish, and German by members of each country’s research team — native Portuguese speakers in Brasil, Spanish speakers in Colombia, and German speakers in Germany. Subsequently, the guide was back-translated into English to ensure accuracy and cosistency. The interview guide included participants’ socioeconomic characteristics and open-ended questions on their work-related experiences during the COVID-19 pandemic, including stressful situations, workload, resource availability, emotional experiences, and support received from supervisors, colleagues, policy makers, and society, as well as the impacts of vaccines availability. The full guide is attached in the Supplementary material 1.

In Brazil, primary care workers from four centers in São Paulo, the COVID-19 epicenter in Brazil (15), participated in four focus group sessions with a total of 45 participants. The sessions lasted about 100 min each and were audio-recorded. Data collection took place from June 20 to September 4, 2022.

In Colombia, we invited participants from primary, secondary, and tertiary healthcare services in Medellín, Bogotá, and Cali, which were heavily impacted by COVID-19 (16). We conducted three focus groups with 3–4 participants each. Sessions lasted about 90 min and took place between June 20 and September 15, 2022.

In Germany, we invited participants from hospitals and clinics in Emden. We conducted three focus groups, each lasting about 90 min, between April 28, 2022, and July 28, 2022.

### Data analysis

Focus groups were audio-recorded and transcribed verbatim. The analysis involved multiple readings of the transcripts to understand the conveyed meaning, identifying significant phrases, and noting key points expressed by participants. The text was divided into meaning units, which were condensed while preserving their core meaning. Codes were then formulated from these condensed units, and grouped into categories (17). In each country, the meanings were formulated and validated by the research team to reach a consensus.

### Researchers’ characteristics and reflexivity

In Brazil, focus groups were conducted in Portuguese by a family physician with experience in mental health and primary care research. In Colombia, the focus groups were conducted in Spanish by a psychologist with experience in mental health. In Germany, they were conducted in German by a sociologist with expertise in qualitative methods.

### Ethics

Ethics approval was obtained from the institutional review boards in Brazil (IRB of the Department of Healthcare of the municipality of São Paulo, approval number 4.160.385), Colombia (Bioethics Committee of the National School of Public Health, approval number 21030002-00104-2022), and Germany (Commission for Impact Assessment and Ethics of the University of Applied Sciences Emden/Leer, Germany, approval number is 2022_MIND_01). All participants were assured of privacy and confidentiality and signed an informed consent before participation. Confidentiality was maintained by using identifiers (e.g., physician P1, nurse N1; nursing assistant NA1, NA2; administrative staff AS1, community health worker CHW1, etc.) instead of names.

## Results

### Sample

Our sample consisted of 63 HCWs, from Brazil (*n* = 45), Colombia (*n* = 9), and Germany (*n* = 9). The majority were women (82%, *n* = 52), and most were between the ages of 31 and 50 years (*n* = 42; 66.7%). The HCWs included were from primary care, secondary care, and hospital settings. Regarding job types, 47.7% were clinical staff (including physicians, nurses, nursing assistants, psychologists, dentists, nutritionists, and pharmacists), 36.5% were CHWs, and 15.8% were administrative staff (Table 1).

**Table 1:** Participants’ characteristics in Brazil, Colombia, and Germany (*n* = 63).

	Total *n* = 63	Subjects in Brazil *n* = 45	Subjects in Colombia *n* = 9	Subjects in Germany *n* = 9
	*n* (%)	*n* (%)	*n* (%)	*n* (%)
Gender				
Female	52 (82.5)	41 (91.0)	6 (66.6)	5 (55.6)
Male	11 (17.5)	4 (9.0)	3 (33.3)	4 (44.4)
Age group (years)				
20 to 30	11 (17.5)	9 (20.0)	2 (22.2)	-
31 to 40	24 (38.1)	18 (40.0)	4 (44.4)	2 (22.2)
41 to 50	18 (28.6)	16 (35.5)	-	2 (22.2)
51 or more	10 (15.8)	2 (4.5)	3 (33.3)	5 (55.6)
Healthcare facility				
Primary care	52 (82.5)	45 (100.0)	7 (77.7)	-
Hospital	9 (14.3)	-	-	9 (100)
Secondary care	2 (3.2)	-	2 (22.2)	-
Job type				
Physicians	7 (11.1)	4 (9.0)	2 (22.2)	1 (11.1)
Nurses/Nursing assistants	18 (28.6)	10 (20.0)	5 (55.5)	3 (33.3)
Community health workers	23 (36.5)	23 (53.0)	-	-
Administrative staff	10 (15.8)	4 (9.0)	2 (22.2)	4 (44.4)
Other clinical staff*	5 (8.0)	4 (9.0)	-	1 (11.1)

*psychologists, dentists, nutritionists, and pharmacists.

**Figure 1:**
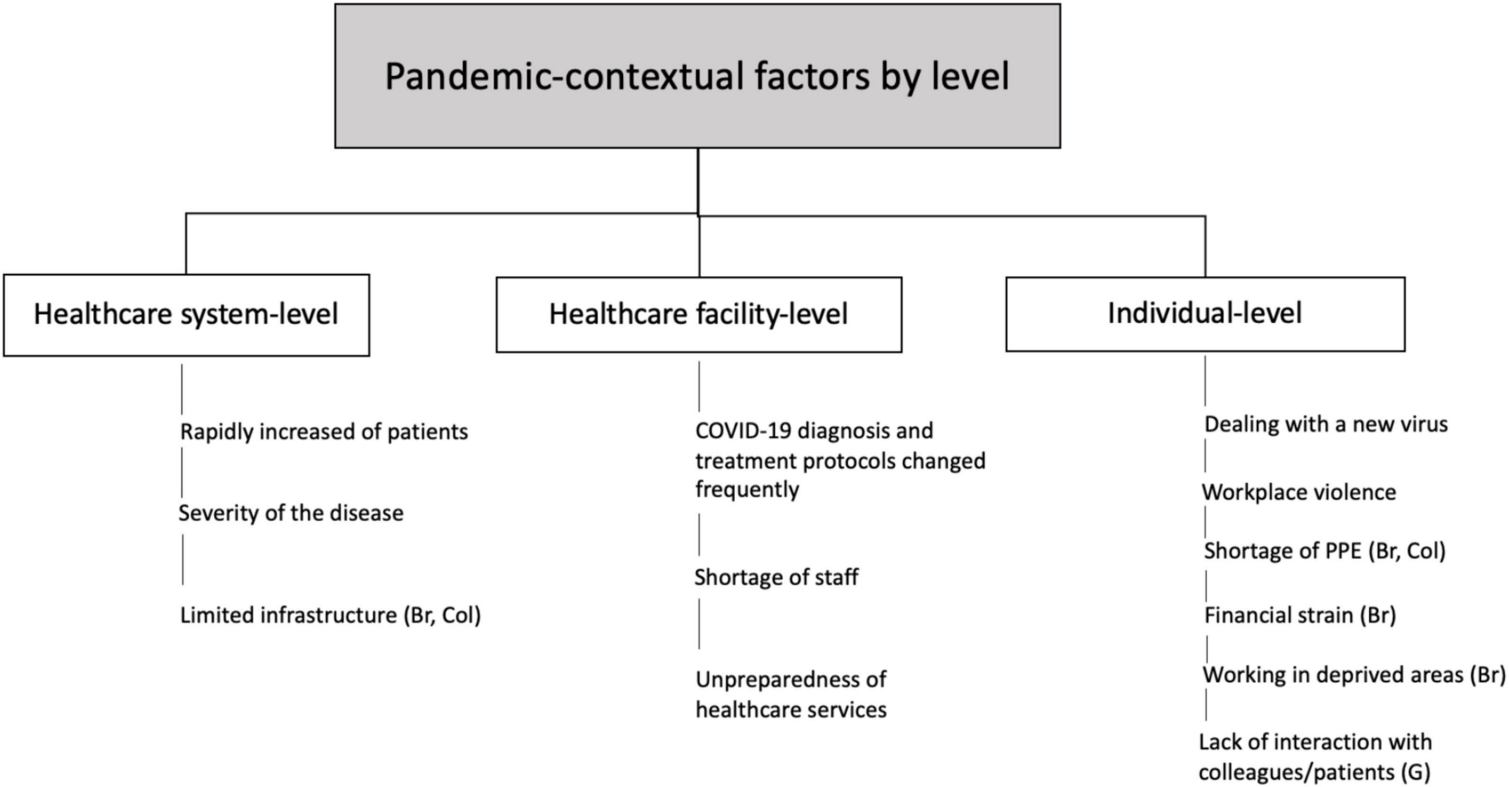
Pandemic-contextual factors that affected HCWs’ mental health during COVID-19 in Brazil (Br), Colombia (Col), and Germany (G).

The pandemic-contextual factors that impacted HCWs’ mental health were categorized into three levels: healthcare system, facility, and individual (Figure 1). Common factors reported across all three countries included:

1. *Healthcare system level*: Rapid increase in patients, and severity of the disease.
2. *Facility Level*: Frequent updates to COVID-19 protocols, shortage of staff, and lack of preparedness in healthcare services.
3. *Individual level*: Dealing with a new virus, and workplace violence.
4. Country-specific differences included:
5. *Brazil and Colombia*: Healthcare system’s limited infrastructure, and shortage of PPE.
6. *Brazil*: working in deprived areas, and financial strain.
7. *Germany*: lack of interactions with colleagues as a significant stressor.

### Healthcare system-level

#### Rapid increase of patients

The exponential increase in the number of patients and work demands led HCWs to experience both physical and mental symptoms.

*Br1 “Before the pandemic, we assisted about 30 non-scheduled patients per day. This number surged to 110 per day during the pandemic. Some arrived critically ill with severe dyspnea, overwhelming our ability to provide adequate care. After the second wave, I became stressed, and impatient, sometimes crying in the bathroom. I needed sick leave due to panic attacks.” (N5)*

*Col1 Overcrowding, because all the gynecology and delivery room patients from the entire sub-network are being received there, so the patients were piled up. (NA 2)*

*G1 The wave really came very quickly, so within a week we really had to increase the staff so much that we went from one person per shift to six people per shift. And actually, it went up and down all the time. (N1)*

#### Severity of the disease

The severity of COVID-19 symptoms resulted in a high influx of patients needing ventilatory support and critical care. HCWs had to adapt their resources to meet their patients’ needs as effectively as possible.

*Br1 Before the pandemic, patients needing critical care were rare. Suddenly, we were overwhelmed, like an emergency room, frequently calling ambulances to transfer patients. Severe dyspnea cases became common. We had to use an oxygen source intended for one patient to support four. (P1)*

*Col1 The stress of putting on all that PPE and dealing with critically ill patients was immense. Many times, you run the risk. I even asked my colleagues to remove my mask while performing cardiac massage because I couldn’t breathe. It was a challenging situation beyond our control. (NA3)*

*G1 The focus on intensive care was overwhelming. Although I come from an intensive care background, and have always been dedicated to it, the reality changed during the third, fourth, and fifth waves. Most of the burden shifted from intensive care units to regular nursing wards, where the distinction between the two became less clear. (AS3)*

#### Limited infrastructure

In Brazil and Colombia, participants reported stressful situations due to a lack of medication and insufficient critical care services to adequately address patients’ needs.

*Br1 We did not have enough oxygen points in our building to meet patient demands, or enough hospital beds to transfer critically ill patients. For the first time, we had to stay in the primary care unit overnight to care for a patient. Our healthcare system was not responding to patients’ demands. It failed to support the population, to support us. (AS2)*

*Col1 Overcrowding, because all the gynecology and delivery room patients from the entire sub-network are being received there, so the patients were piled up. (NA2)*

### Healthcare facility-level

#### COVID-19 diagnosis and treatment protocols changed frequently

Frequent updates to diagnosis and treatment protocols increased HCWs’ stress. They experienced feelings of insecurity, fear of inadequate self-protection, and being overwhelmed by the constant training on new protocols.

*Br1 When we get used to the new protocol, another protocol appears and then another one. I felt exhausted … I asked myself, ‘is this protocol correct?’ The information about the disease was confusing—for example, isolation recommendations changed from 14 days to 7 days. I feared getting sick and dying. (NA)*

*Col1 The protocol, the previous protocols—I’ll be straightforward. The fear of not following the procedures precisely was constant, I could cause harm, kill myself, or kill someone dear to me. (NA 5)*

*G1 We worked across different settings with varying PPE requirements—medical masks in one place, FFP2 masks in another. It would have been easier if there was consistency in the type of masks used. The constant switching and carrying around different types of masks added to the stress. (N1)*

#### Shortage of staff

Many HCWs contracted COVID-19 and others were absent from work due to mental health problems, which increased the workload for the remaining team members. The shortage of staff to handle the work demands contributed to heightened stress and anxiety.

*Br1 We faced a shortage of workers to manage the demand because many of us contracted the virus. Several colleagues were sick simultaneously, creating a desperate situation. (Ph1)*

*Col1 Out of 20 people in that service, seven were incapacitated, two were under suspicion of COVID-19, and one was dealing with severe depression. This left just 10 of us to handle the responsibilities normally managed by a team of 25. We had to work longer hours and perform tasks outside our usual roles. (P2)*

*G1 After multiple COVID-19 waves, we are increasingly seeing the effects on our staff. What worries us more and more is that with rising patient numbers, staff members are not just missing work for six, or seven days, but are instead absent for extended periods due to illness. (AS1)*

#### Lack of preparedness of healthcare services

Participants expressed their concern about the unpreparedness of healthcare facilities to properly respond to the COVID-19 crisis, especially in managing limited resources for patients with severe symptoms.

*Br1 Suddenly, we began attending to patients with severe dyspnea, and had to provide respiratory support, including mechanical ventilation, in a primary care center. We lacked medication, sedatives, and necessary equipment. It was a terrifying situation. (P 4)*

*Col1 Hospitals did everything they could with limited resources available. They mobilized efforts to set up artificial respirators and increase ICU beds. Even hospitals that were not fully prepared had to adapt quickly to manage the influx of patients. It was a tremendous effort to scale up their capabilities under such pressure. (P2)*

*G1 We initially hoped the situation would be short-term, similar to the EHEC [Enterohemorrhagic Escherichia Coli] crisis. We canceled all meetings and didn't even start digitizing processes. After six months, we began to realize we couldn’t cancel everything completely, and it became clear that there was no end in sight. (N 1)*

### Individual-level

#### Dealing with a new virus

HCWs highlighted their mental burden in dealing with a new, highly transmissible disease and the lack of available knowledge about COVID-19 treatment. Managing the exponential increase in patient numbers, along with coping with the deaths of patients and colleagues, significantly contributed to their work overload, emotional distress, and mental burden.

*Br1 Too many patients and too much work. I started to become accustomed to the sound of ambulances. Later, a patient we transferred to the hospital passed away. I felt both physically and emotionally exhausted. (N 3)*

*Br2 In our catchment area, there were so many deaths, including people we had been looking after for years. Sometimes, we would see an entire family–father, mother, and child—pass away within a week. I felt heartbroken and hopeless. (CHW 4)*

*Col1 Excuse me if my voice breaks … It was a very difficult time, colleagues in the ICU, and a chief who died there without us being able to help him. It was an intense experience with many painful memories. It will be marked us forever. (NA 1)*

*G1 One of the worst things was that when the COVID patients died, they couldn’t be treated with the same dignity as non-infectious patients. Instead, their bodies had to be placed in black infectious bags. We had to handle these bags and transport them, which was incredibly difficult and distressing. (N3)*

#### Workplace violence

Workplace violence was reported in all three countries. HCWs expressed feelings of injustice, anger, sadness, and fear of experiencing violence again.

*Br1 Most mistreatment stemmed from delays in being assisted. The demand increased, and the staff decreased. Patients had to wait a long time, which led to yelling, insults, or hitting doors. Once, a patient threw a chair at us in the reception area. It affected my emotions and made me feel angry. (AS 4)*

*Col2 The head nurse on the floor said, ‘Guys, we are in alarm.’ She asked us to inform the patients that we were on alert. Is this really happening? At that point, we realized many people were trying to enter the hospital through the emergency area to attack the staff, accusing us of causing deaths and charging for them. (NA 3)*

*G1 Conflicts regarding masks … It's not just relatives or patients; even colleagues lack understanding. We often had to explain why masks were required. It's not only visitors and patients, but even some colleagues who at some point no longer have any understanding and after almost three years really want to get back to their normal lives somehow. (N 1)*

In Brazil, participants also described mistreatment related to COVID-19 vaccines. Both the lack of and the availability of doses led to aggressive behavior. This type of violence introduced a new dimension to workplace violence.

*Br1 Only a few doses of the vaccines were available to vulnerable groups. We explained that everyone had the right to receive the vaccine, but the government did not provide enough doses. People became angry, and made threats. Aggressions toward us became common. (N 1)*

*Br2 We went to patients’ homes to administer the doses. Some refused, claiming that COVID-19 vaccines were a 'strategy to kill elderly people' and 'not created by the Lord.' We were risking our own safety to give them the vaccine. I had mixed feelings—feeling sorry for them, but also angry. It seemed so unfair; I even considered giving up. (CHW 10)*

#### Shortage of PPE

In Brazil and Colombia, insufficient access to PPE increased HCWs’ fear of contracting and transmitting COVID-19, becoming a significant source of stress. In Brazil, the availability of PPE varied across healthcare settings, with primary care experiencing the worst shortages.

*Br1 Before the pandemic, we only had N95 masks available for attending patients with tuberculosis. During the COVID-19 pandemic, there were not enough masks for hospital workers; and it was even worse in primary care. We faced not only a shortage of PPE but also low-quality supplies. I felt inadequately protected. (N 2)*

*Col1 The most distressing situation for me was when biosecurity elements started running out at the hospital. First, gloves and gowns were missing. It felt like trying to defend oneself with just your fingernails and avoid bringing the virus home as much as possible. (NA 4)*

#### Financial strain

In Brazil, CHWs reported experiencing financial strain during the pandemic. Their salaries were reduced due to sick leave from COVID-19 and the economic impact of the pandemic, making it difficult to cover expenses. This financial strain had consequences for their mental health.

*Br1 My recovery from COVID-19 took longer than expected, and I had to extend my sick leave by more than 15 days. My salary was reduced to the point where I couldn’t even pay for cooking gas. It affected my entire family, and I began treatment for depression. (CHW 8)*

*Br2 In the first year of the pandemic, with social distancing measures, my husband and son lost their jobs. My salary wasn’t enough to cover rent or provide sufficient food. By a stroke of divine providence, we received monthly donations from the church. (CHW 12)*

#### Working in deprived areas

In Brazil, HCWs highlighted their experiences dealing with the economic consequences of the pandemic on vulnerable populations. As primary care workers, they witnessed the harsh realities faced by these communities. Many patients struggled to secure enough food.

*Br1 I had never encountered this before the pandemic. I had to deal with patients in my area who were starving. It was heartbreaking to see patients crying because they had nothing to eat. During home visits, a woman was embarrassed to tell us she was losing weight because she had no food, but her neighbor informed the CHW. We asked for help from community institutions that could provide food. (NA 3)*

#### Lack of interaction with colleagues

In Germany, participants described the lack of face-to-face interaction with colleagues as a significant stressor. The COVID-19 crisis altered their work routines, limiting their interactions and preventing opportunities to discuss stressful situations, which added to their mental strain.

*G1 What really suffered was the normal social exchange … sitting together in the common room at the end of a shift, having a coffee, or just talking about what we’ve experienced. Hygiene measures prevented these interactions. It put a strain on the staff (AS2)*

## Discussion

Our data showed that HCWs faced common pandemic-related factors in all three countries studied. At the healthcare system level, these included a rapid influx of patients and severe cases. At the facility level, frequent protocol changes, staff shortages, and unprepared services were significant. At the individual level, challenges included dealing with a new virus, work overload, and workplace violence. In Brazil and Colombia, limited healthcare infrastructure and PPE shortages were key issues. Additionally, in Brazil, financial strain and working in underserved areas impacted HCWs’ mental health, while in Germany, a notable stressor was the lack of colleague interaction.

The pandemic brought new stressors for HCWs, with the high transmissibility of COVID-19 causing a rapid surge in patients needing care. This increase, along with severe symptoms and a demand for critical care, overwhelmed healthcare systems and significantly impacted HCWs’ mental health (8, 18, 19). The lack of preparedness in healthcare systems to handle COVID-19 increased HCWs’ workload and stress, particularly in low-and middle-income countries where the pandemic overwhelmed resources (20). Our data showed that HCWs in Brazil and Colombia specifically noted limitations in their healthcare systems, such as shortages of critical care beds, medications, and oxygen. These challenges led to feelings of anguish, despair, and helplessness, worsening their mental burden. Inadequate resources to care for patients heightened distress among HCWs (8, 20, 21), which was further intensified by witnessing numerous patient deaths. The inability to provide adequate care, including insufficient pain medication, and seeing patients die in undignified manners, has been associated with severe distress and post-traumatic stress disorder in HCWs (21, 22).

Dealing with a new virus resulted in frequent changes to diagnosis and treatment protocols, increasing HCWs’ responsibilities and requiring them to learn new tasks and keep up with evolving guidelines (23, 24). Additionally, increased paperwork, longer shifts, and extended working hours due to staff shortages from COVID-19-related sick leave elevated workloads and negatively impacted HCWs’ mental health (8). The uncertainty, insecurity, and challenging practices, coupled with healthcare the lack of preparedness in healthcare services to provide a safe workplace and adequate support for managing a highly transmissible disease, heightened HCWs’ fears of becoming infected and potentially transmitting the virus to loved ones (8, 25). The shortage of PPE made HCWs feel unsafe, a concern particularly emphasized by participants in Brazil and Colombia. PPE shortages in Brazil and Colombia likely reflect resource disparities observed in LMICs, unlike Germany’s more robust supply chains.

Workplace violence toward HCWs has been a global concern even before the pandemic (26, 27). However, the pandemic introduced new elements to this issue (28). The lack of preparedness in health systems to meet patients’ needs exacerbated the problem (4), potentially increasing mistreatment of HCWs. Perpetrators of violence were most commonly relatives of COVID-19 patients, with the main reason being refusal to admit patients and the deaths of COVID-19 patients (3). Additionally, misinformation about COVID-19 on social media heightened public fear of HCWs as potential sources of infection, leading to conflicts (29). Ineffective government communication and anti-science measures worsened outcomes, including increasing cases and deaths, vaccine hesitancy, and prolonging the pandemic, all of which contributed to heightened stress among HCWs (30). In Brazil, HCWs reported mistreatment related to misinformation about COVID-19 vaccines, with the Brazilian government’s role in spreading misinformation potentially contributing to this mistreatment (31). This context introduced new elements to the workplace violence experienced before the pandemic.

Our data also included CHWs in Brazil, who are part of the Family Health Program—one of the largest primary care programs globally, covering 132 million people nationwide. Trusted members of their local communities, CHWs played a crucial role in the healthcare system’s response to COVID-19. Their responsibilities included guiding protective measures, providing accurate information about transmission, identifying vulnerable groups, following up with patients, and addressing misinformation and stigma surrounding COVID-19 (32, 33). In our study, community health workers reported financial strain linked to long sick leaves due to COVID-19, which reduced their monthly salaries, and increased their risk of mental burden (34). Despite their crucial role during global crises, especially in low-and middle-income countries (LMICs), the working conditions for community health workers remain substandard (35). Providing financial support for community health workers during sick leave, along with implementing community food programs, could help mitigate these stressors in future crises in Brazil.

Primary care workers faced additional challenges, including managing the economic consequences of COVID-19 in deprived areas with higher prevalence of multimorbidity and long COVID-19 (36–38). This work-related context may have impacted their mental health (39, 41).

Participants in Germany specifically highlighted how the lack of interaction with colleagues negatively impacted their mental health. In this regard, previous studies have found that social interactions and support from colleagues during the pandemic were associated with a lower risk of mental health problems (40, 42).

### Strengths and limitations

This study presents several strengths. The qualitative cross-country design enabled an in-depth exploration of HCWs experiences across different sociocultural and healthcare system contexts. The inclusion of HCWs from primary care settings, including community healthcare workers, helped address important gaps in previous research. Conducting interviews later in the pandemic allowed for reflections after 2 years of COVID-19, offering a more comprehensive view of sustained stressors. In addition, our findings provide starting points for analyzing quantitative data from the countries that were part of the study.

However, several limitations should be acknowledged. First, as a qualitative study, the results are not generalizable, although they provide rich contextual insights into the Brazilian, Colombian, and German healthcare systems. Given the lack of previous comparative studies involving these three countries, our findings offer valuable preliminary insights. Second, memory bias may have affected participants’ recollections of early pandemic experiences. We sought to mitigate this by emphasizing recurring themes and consistent narratives reported throughout 2022. Third, sample sizes varied across countries. The smaller number of participants from Colombia and Germany—compared to Brazil—may reflect differences in willingness to participate. This imbalance limits analytical comparisons and subgroup analyses. Additionally, the sample distribution across gender, age groups, facility types, and professional roles was uneven, which may further constrain the generalizability of findings. Despite these challenges, we used purposive sampling to ensure heterogeneity in each country, and we conducted in-depth analyses to capture the complexity of participants’ experiences. Nevertheless, the greater volume of data from Brazil may have led to a disproportionate emphasis on Brazil-specific themes. Finally, participants were drawn from selected regions within each country, potentially excluding perspectives from remote or underserved areas.

Future qualitative studies with larger, more balanced, and regionally diverse samples are needed to validate and expand upon our findings.

## Conclusion

Our results revealed that variations in experiences among HCWs in the three countries highlighted work-related inequities across healthcare settings. Primary care workers faced unique stressors due to their community-based roles. At the healthcare system level, healthcare structures should be flexible enough to address a rapid increase in patients and severity of the pandemic disease, for example through designated reserve staffing pools or mutual aid agreements between regions depending on the pandemic load. At the healthcare facility level, living protocol repositories should be established to enable frequent updates to pandemic disease protocols without losing focus, flexible staffing models such as cross-functional staff teams (e.g., a mix of ICU and non-ICU personnel with *ad hoc* training) to make better use of available resources in times of crisis and address shortage of staff, and. At an individual level, easily accessible information on new developments in the pandemic situation should be provided, as well as psychosocial counseling for staff at all levels of the hierarchy to address the mental health burden of healthcare workers (HCWs), and violence-prevention training to empower all staff and address physical and psychological violence. Country-specific recommendations, based on the findings of our study, are improving healthcare system’s infrastructure and shortage of PPE in Brazil and Colombia, improving living, working and economic situation of HCWs living in vulnerable situations (e.g., working in deprived areas) in Brazil, and promoting interaction between colleagues (e.g., peer support and informal conversations) and improving digitalization in the health care systems in Germany.

## Data Availability

The datasets presented in this study can be found in online repositories. The names of the repository/repositories and accession number(s) can be found in the article/Supplementary material.
