# Supplementary material for: Commonalities and differences in healthcare workers’ perceptions of mental burden in Brazil, Colombia, and Germany during the COVID-19 pandemic: a qualitative cross-country study
Source: Front Public Health. 2025 Jul 15;13:1542494. doi: 10.3389/fpubh.2025.1542494 (PMC12303911; doi:10.3389/fpubh.2025.1542494)
Supplement: Supplementary file 1 [file Data_Sheet_1.pdf]

Supplementary material 1

Part 1: Socio-demographic questions

Age: _________

Gender: _______

Highest level of education attained:

Occupation and position: __________

Workplace: ___________

How long have you been working at your current workplace?

Are you working at your current workplace during the pandemic since?

Do you have children, older adults, or other people you care for living at home?

Part 2 -Healthcare workers' experiences during the COVID-19 pandemic

2.1 General experience

Please, tell me about your experience during the COVID-19 pandemic.

What was the most stressful situation you experienced during the pandemic?

What was the most helpful situation that helped you cope with the stress during the pandemic?

2.2 Work-related experiences

What do you think were the most stressful work-related aspects during the COVID-19 pandemic?

What do you think were the most helpful work-related aspects during the COVID-19 pandemic?

Could you tell us about mental health during the COVID-19 that you noticed among healthcare workers?

Did you feel that you were supported by superiors, colleagues, policy makers and society during the COVID 19 pandemic?

Did you notice any changes in people's behavior towards each other during the pandemic?

Did the work environment change after the vaccine was available for healthcare workers?
